# Associations between apparent diffusion coefficient (ADC) and KI 67 in different tumors: a meta-analysis. Part 2: ADC_min_

**DOI:** 10.18632/oncotarget.24006

**Published:** 2018-01-04

**Authors:** Alexey Surov, Hans Jonas Meyer, Andreas Wienke

**Affiliations:** ^1^ Department of Diagnostic and Interventional Radiology, University of Leipzig, Leipzig, Germany; ^2^ Institute of Medical Epidemiology, Biostatistics, and Informatics, Martin-Luther-University Halle-Wittenberg, Halle (Saale), Germany

**Keywords:** diffusion weighted imaging, ADC, KI 67

## Abstract

The purpose of this part of the meta-analysis was to summarize data regarding associations between minimum apparent diffusion coefficient (ADC_min_) and KI 67 in different tumors.

MEDLINE library was screened for associations between ADC_min_ and KI 67 in different tumors up to April 2017. Overall, 23 studies with 944 patients were identified. Associations between ADC and KI 67 were analyzed by Spearman's correlation coefficient.

The pooled correlation coefficient between ADC_min_ and KI 67 for all included tumors was ρ = -0.47. In detail, the correlation coefficients for separate tumors were as follows: cerebral lymphoma: ρ = –0.61 (95% CI = [–0.82; –0.41]); cervical cancer: *ρ* = –0.56 (95% CI = [–0.68;–0.43]); pituitary adenoma: *ρ* = –0.55 (95% CI = [–1.31; 0.22]); glioma: *ρ* = –0.40 (95% CI = [–0.55; –0.24]); breast cancer: *ρ* = –0.37 (95% CI = [–0.74; –0.01]); meningioma, *ρ* = –0.15 (95% CI = [–0.38; 0.07]).

## INTRODUCTION

Apparent diffusion coefficient (ADC) is a quantitative parameter of water diffusion in tissues [1]. Previously, numerous studies investigated associations between ADC and several histopathological features in different tumors [2–5]. Some reports indicated that ADC can predict proliferation activity and, therefore, behavior of several malignancies [2, 3, 5]. As already mentioned, ADC can be divided into three sub-parameters: ADC minimum or ADC_min_, mean ADC or ADC_mean_ and ADC maximum or ADC_max_ [5]. As shown in the part 1 of this meta-analysis, several tumors showed different inverse correlations between ADC_mean_ and KI 67 [6]. Overall, the calculated correlation coefficients ranged from –0.22 in breast cancer to –0.62 in ovarian cancer [6].

There were studies, which showed that ADC_min_ had stronger correlations with KI 67, and can better reflect proliferation potential of malignant lesions [7, 8]. However, the reported data were based on small number of investigated tumors/patients.

The purpose of this part of the meta-analysis was to provide evident data regarding associations between minimum ADC (ADC_min_), and KI 67 in different tumors.

## RESULTS

Overall, the identified 22 studies [7–28] contained data about associations between ADC_min_ and KI 67 for 944 patients (Table 1).

**Table 1:** Tumor types involved into the meta-analysis

Diagnosis	*n*	%
Different breast tumors	200	34.33
Glioma	144	15.25
Cervical cancer	117	12.39
Lung cancer	93	9.85
Meningioma	72	7.63
Pituary adenoma	50	5.30
Cerebral lymphoma	49	5.19
Prostatic cancer	29	3.07
Neuroendocrine tumor	22	2.33
Thyroid cancer	14	1.48
Head and neck cancer	11	1.17
Ganglioglioma	10	1.06
Neurocytoma	9	0.95
Total	944	100

**Figure 1:**
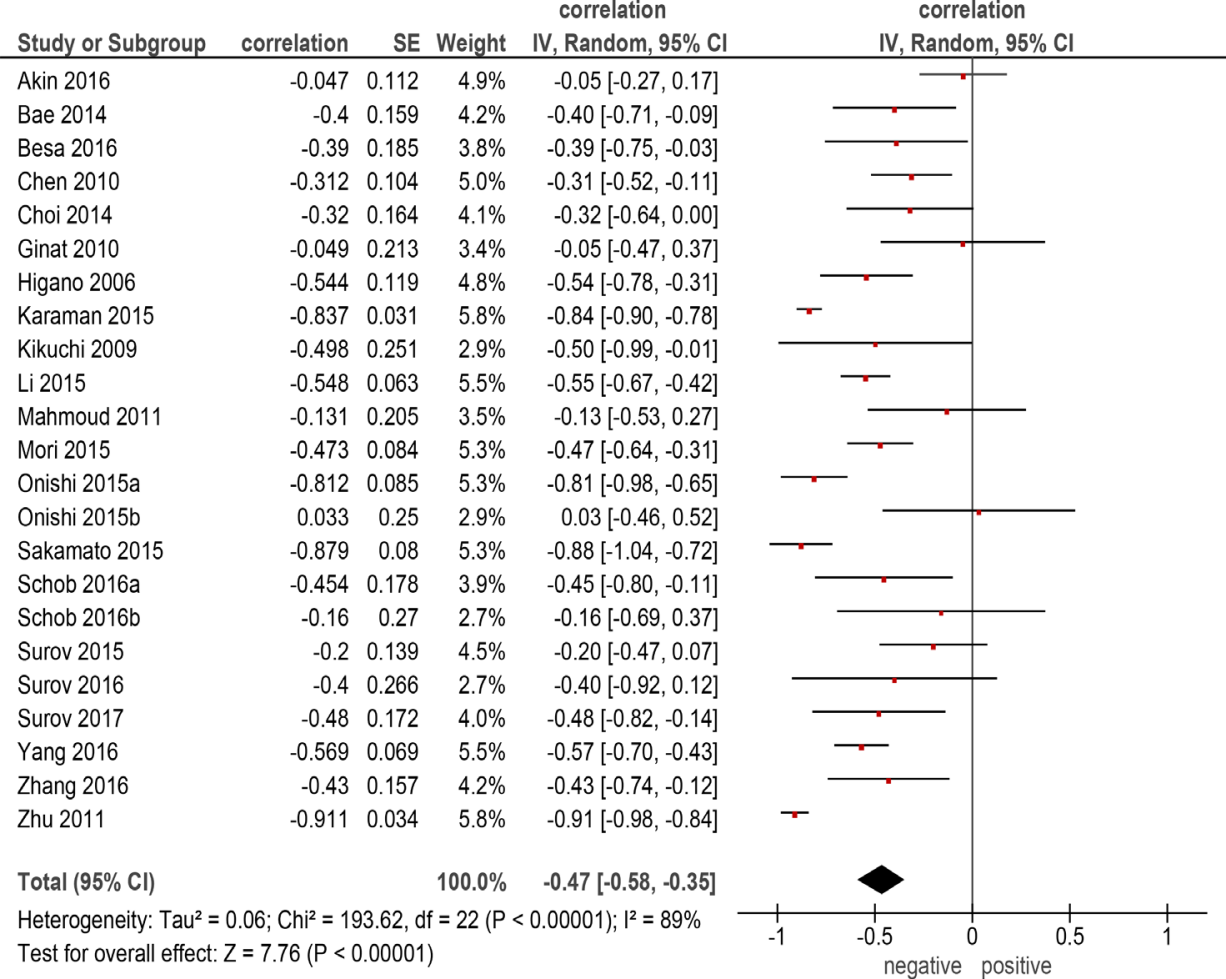
Forest plots of correlation coefficients between ADC_min_ and KI 67 in all included studies (*n* = 22)

The pooled correlation coefficient for all patients (Figure 1) was –0.47 (95 % CI = [–0.58; –0.35]), heterogeneity Tau^2^ = 0.06, Chi^2^ = 193.62, df = 22 (*P* < 0.00001), I^2^ = 89 %, and test for overall effect Z = 7.76 (*P* < 0.00001).

**Figure 2:**
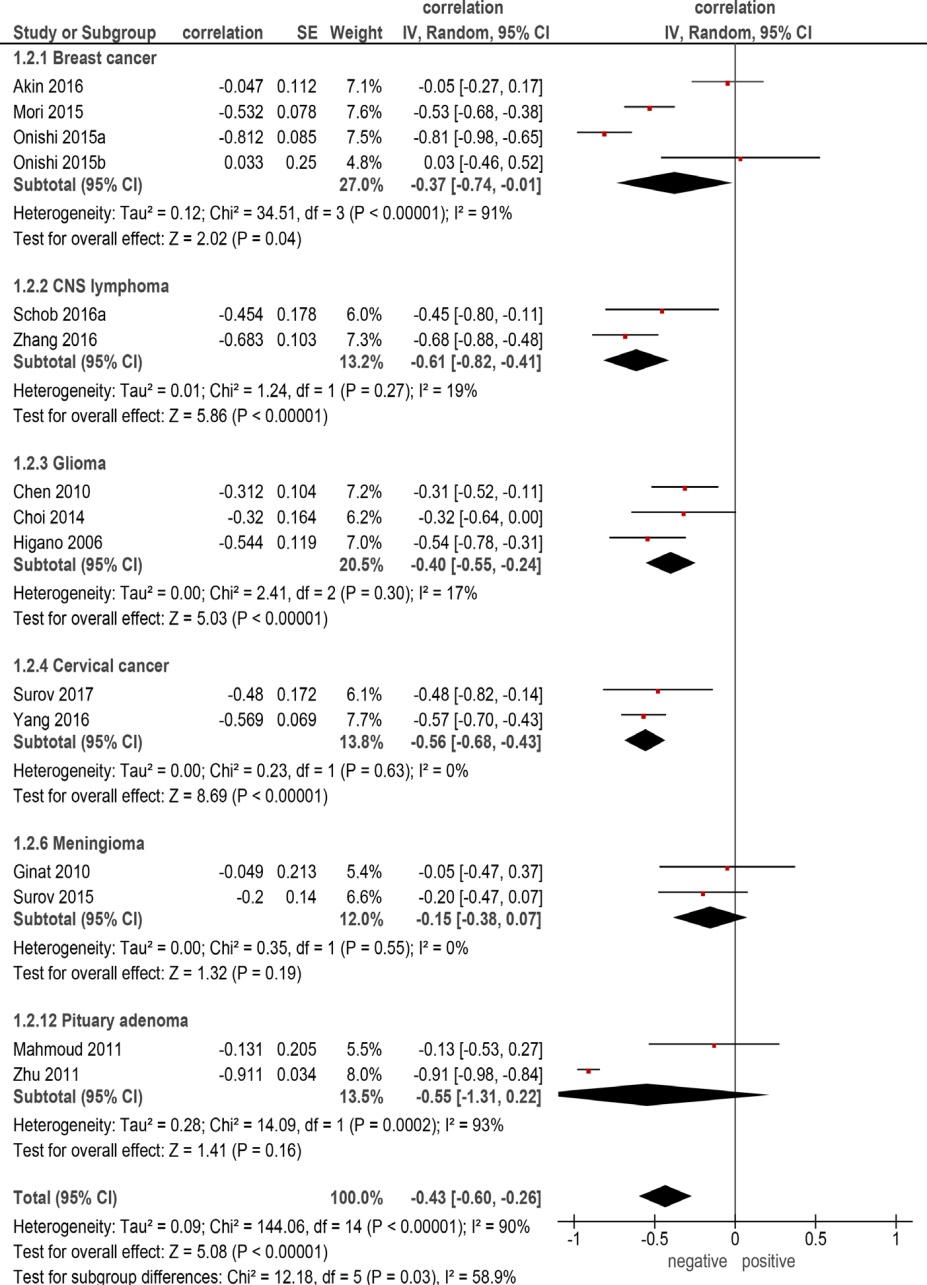
Forest plots of correlation coefficients between ADC_min_ and KI 67 in different primary tumors

On the next step correlation analysis for every identified entity was performed. Thereby, only primary tumors with more than two reports were included into the analysis. There were 6 entities with 632 patients (Table 2). The calculated correlation coefficients were as follows (Figure 2): -cerebral lymphoma: *ρ* = –0.61 (95% CI = [–0.82; –0.41]); -cervical cancer: *ρ* = –0.56 (95% CI = [–0.68;–0.43]); -pituitary adenoma:*ρ* = –0.55 (95% CI = [–1.31; 0.22]); -glioma: ρ = –0.40 (95% CI = [–0.55; –0.24]); - breast cancer: *ρ* = –0.37 (95% CI = [–0.74; –0.01]); -meningioma, *ρ* = –0.15 (95% CI = [–0.38; 0.07]).

**Table 2:** Tumor entities included into the subgroup analysis

Diagnosis	*n*
Breast cancer	200
Glioma	144
Cervical carcinoma	117
Meningioma	72
Pituary adenoma	50
Cerebral lymphoma	49

## DISCUSSION

### The present meta-analysis summarizes data about associations between ADC_min_ and KI 67 in different tumors

Previously, some investigations focused on relationships between ADC and histopathology, such as cell count and/or proliferation potential, in several tumors [2, 5]. However, the reported data were inconsistent: while some authors mentioned that ADC fractions can be associated with cellularity and KI 67, others did not confirm this finding [5, 7, 8]. Our previous meta-analysis regarding correlation between ADC_mean_ and tumor cellularity showed that several tumors have different associations between the investigated parameters [29]. In detail, the calculated correlation coefficients ranged significantly and were as follows: *ρ* = –0.25 in lymphoma, *ρ* = –0.45 in meningioma, *ρ* = –0.48 in breast cancer, *ρ* = –0.53 in renal cell carcinoma, *ρ* = –0.53 in head and neck squamous cell carcinoma, *ρ* = –0.56 in prostatic cancer, *ρ* = –0.57 in uterine cervical cancer, *ρ* = –0.63 in lung cancer, *ρ* = –0.64 in ovarian cancer, and *ρ* = –0.66 in glioma [29]. Almost similar results were also identified for associations between ADC_mean_ and KI 67 in the part 1 of the present work [6]. Because of these findings it can be postulated that ADC_mean_ does not reflect cellularity and proliferation potential in all tumors and tumor-like lesions as assumed previously.

According to some authors, another ADC parameter, namely ADC_min_ has been reported to be more sensitive in prediction of cell count and proliferation activity than ADC_mean_ [2, 7, 8]. However, a recent meta-analysis showed that ADC_min_ did not better correlate with tumor cellularity than ADC_mean_ [30].

### There were also inconsistent data about correlation between ADC_min_ and proliferation activity

As seen, in the present analysis, ADC_min_ correlated moderately with KI 67 expression in overall sample. The calculated correlation coefficient (*ρ* = –0.47) was almost similar to those reported for ADC_mean_ (*ρ* = –0.44). However, for the identified tumor entities, it was different in comparison with the coefficients for ADC_mean_. So, in breast cancer, ADC_min_ correlated stronger with KI 67 (*ρ* = –0.37) than ADC_mean_ (*ρ* = –0.22) [6], although the identified associations were slightly. Also in pituitary adenoma, and cerebral lymphoma, ADC_min_ tended to be better in comparison to ADC_mean_: *ρ* = –0.56 vs *ρ* = –0.44 [6], and *ρ* = –0.61 vs *ρ* = –0.55, respectively [6]. On the other hand, in glioma and meningioma, ADC_min_ did not better correlate with KI 67 expression than ADC_mean_: *ρ* = –0.40 vs *ρ* = –0.51 [6], and *ρ* = –0.15 vs *ρ* = –0.43 [6], respectively.

The exact cause of our findings is unclear. They supported previous suggestions that different ADC fractions reflect different histopathological features [2]. Obviously, there is no general rule regarding ADC parameters and tumor proliferation, i.e. for some tumors ADC_min_ and for other ADC_mean_ predicts better proliferation potential.

Also for this part of the meta-analysis, already the mentioned limitations [6] do apply: only 6 named above tumor entities were involved into the work. For other malignancies and tumor-like lesions no data could be provided. In addition, the number of patients in the groups of pituitary adenoma, cerebral lymphoma, and meningioma was very small that questions the validity of the estimated correlation coefficients.

In conclusion, there are different inverse correlations between ADC_min_ and KI 67 in several tumors. In comparison with ADC_mean_, ADC_min_ seems to correlate better with proliferation activity in breast cancer, cerebral lymphoma, and pituitary adenoma.

In meningioma and glioma, however, ADC_mean_ reflects better tumor proliferation than ADC_min_.

## MATERIALS AND METHODS

### Data acquisition and proving

The search strategy and data acquisition are described precisely in the part 1 of the meta-analysis [6]. For this part, only data regarding associations between ADC_min_ derived from diffusion weighted imaging (DWI) and expression of KI 67 in different tumors and tumor-like lesions were collected. The Preferred Reporting Items for Systematic Reviews and Meta-Analyses statement (PRISMA) was used for the research [31].

Overall, 22 studies were included into the present analysis [7–28]. The following data were extracted from the literature: authors, year of publication, number of patients, tumor type, and correlation coefficients.

### Meta-analysis

The methodological quality of the 23 studies was independently checked by two observers (A.S. and H.J.M.) using the Quality Assessment of Diagnostic Studies (QUADAS) instrument according to previous descriptions [32, 33]. The results of QUADAS proving is given in Table 3.

**Table 3:** Methodological quality of the involved 23 studies according to the QUADAS criteria

QUADAS criteria	Yes (%)	No (%)	Unclear (%)
Patient spectrum	23 (100)		
Selection criteria	20 (86.96)		3 (13.04)
Reference standard	23 (100)		
Disease progression bias	23 (100)		
Partial vertification bias	23 (100)		
Differential vertification bias	23 (100)		
Incorporation bias	23 (100)		
Text details	23 (100)		
Reference standard details	23 (100)		
Text review details	12 (52.18)	3 (13.04)	8 (34.78)
Diagnostic review bias	15 (65.22)	3 (13.04)	5 (21.74)
Clinical review bias	23 (100)		
Uninterpretable results	23 (100)		
Withdrawls explained	23 (100)		

Associations between ADC_min_ and KI 67 were analyzed by Spearman’s correlation coefficient. The reported Pearson correlation coefficients in some studies were converted into Spearman correlation coefficients as described previously [34].

The meta-analysis was undertaken by using RevMan 5.3 (*Computer program, version 5.3. Copenhagen: The Nordic Cochrane Centre, The Cochrane Collaboration, 2014*)*.* Heterogeneity was calculated by means of the inconsistency index I^2^ [35, 36]. In a subgroup analysis, studies were stratified by tumor type. Furthermore, DerSimonian and Laird random-effects models with inverse-variance weights were used without any further correction [37].
